# CONNECTING THE DOTS: COLLABORATIVE LEARNING AMONG OLDER ADULTS, STUDENTS, AND INSTRUCTORS

**DOI:** 10.1093/geroni/igad104.0084

**Published:** 2023-12-21

**Authors:** Caitlin Reynolds

**Affiliations:** North Carolina State University, Raleigh, North Carolina, United States

## Abstract

Although the aging process is a universal phenomenon, misconceptions regarding late adulthood are prevalent, such as loneliness and dependency, which often lead to negative stereotypes and poor developmental outcomes (Kotter-Grühn, 2015); thus, it is important to minimize these stereotypes in an educational platform. Intergenerational discussions have been shown to be a robust approach in reducing stereotypes (Canedo-García et al., 2017; Golenko et al., 2019); especially if it is used to dismantle “othering” the groups by creating community and collectivist perspectives (D’Antonio, 2020). My goal is to explore how intergenerational discussions can be used in the classroom to facilitate collaborative learning among older adults, students, and instructors. In the Fall 2022 and Spring 2023 semesters, I partnered with the Osher Lifelong Learning Institute (OLLI) to connect undergraduate students with older adults from the community. The course centered around the students’ social innovation project- address how current systems may not adequately support older adults and propose a creative solution. The students interviewed the OLLI members throughout the semester about course content (e.g., their awareness of age-related discrimination and barriers in their community). At the end of the semester, students used their accumulated information from both the OLLI members and the course material to propose their social innovation to the OLLI members. The OLLI members asked questions, made recommendations, and highlighted limitations in the students’ work. I observed collaborative thinking and sharing across both groups during these presentations. Overall, intergenerational discussions were a valuable tool in the classroom.

